# Myeloid DNA methyltransferase3b deficiency aggravates pulmonary fibrosis by enhancing profibrotic macrophage activation

**DOI:** 10.1186/s12931-022-02088-5

**Published:** 2022-06-20

**Authors:** Wanhai Qin, C. Arnold Spek, Brendon P. Scicluna, Tom van der Poll, JanWillem Duitman

**Affiliations:** 1 Center of Experimental and Molecular Medicine, Amsterdam University Medical Centers, University of Amsterdam, Meibergdreef 9, Room G2-130, 1105AZ Amsterdam, The Netherlands; 2 Department of Clinical Epidemiology, Biostatistics, and Bioinformatics, Amsterdam University Medical Centers, University of Amsterdam, Amsterdam, The Netherlands; 3 Division of Infectious Diseases, Amsterdam University Medical Centers, University of Amsterdam, Amsterdam, The Netherlands; 4 Department of Pulmonary Medicine, Amsterdam University Medical Centers, University of Amsterdam, Amsterdam, The Netherlands; 5 Department of Experimental Immunology, Amsterdam University Medical Centers, University of Amsterdam, Amsterdam, The Netherlands; 6 Amsterdam Infection & Immunity, Inflammatory Diseases, Amsterdam, The Netherlands; 7 Department of Applied Biomedical Science, Faculty of Health Sciences, Mater Dei Hospital, Centre for Molecular Medicine and Biobanking, University of Malta, Msida, Malta

**Keywords:** DNA methylation, Pulmonary fibrosis, Dnmt3b, Macrophages, Polarization

## Abstract

**Background:**

Idiopathic pulmonary fibrosis (IPF) is a chronic, progressive and severe disease characterized by excessive matrix deposition in the lungs. Macrophages play crucial roles in maintaining lung homeostasis but are also central in the pathogenesis of lung diseases like pulmonary fibrosis. Especially, macrophage polarization/activation seems to play a crucial role in pathology and epigenetic reprograming is well-known to regulate macrophage polarization. DNA methylation alterations in IPF lungs have been well documented, but the role of DNA methylation in specific cell types, especially macrophages, is poorly defined.

**Methods:**

In order to determine the role of DNA methylation in macrophages during pulmonary fibrosis, we subjected macrophage specific DNA methyltransferase (DNMT)3B, which mediates the de novo DNA methylation, deficient mice to the bleomycin-induced pulmonary fibrosis model. Macrophage polarization and fibrotic parameters were evaluated at 21 days after bleomycin administration. *Dnmt3b* knockout and wild type bone marrow-derived macrophages were stimulated with either interleukin (IL)4 or transforming growth factor beta 1 (TGFB1) in vitro, after which profibrotic gene expression and DNA methylation at the *Arg1* promotor were determined.

**Results:**

We show that DNMT3B deficiency promotes alternative macrophage polarization induced by IL4 and TGFB1 in vitro and also enhances profibrotic macrophage polarization in the alveolar space during pulmonary fibrosis in vivo. Moreover, myeloid specific deletion of DNMT3B promoted the development of experimental pulmonary fibrosis.

**Conclusions:**

In summary, these data suggest that myeloid DNMT3B represses fibrotic macrophage polarization and protects against bleomycin induced pulmonary fibrosis.

**Supplementary Information:**

The online version contains supplementary material available at 10.1186/s12931-022-02088-5.

## Introduction

Lung diseases are the leading cause of death worldwide [1]. Among those diseases, idiopathic pulmonary fibrosis (IPF) is a progressive and fatal lung disease with limited therapeutic options [2, 3]. Despite the availability of anti-fibrotic drugs that confer a small increase in overall survival, disease progression is not halted nor reversed by current treatment modalities [4, 5]. Most IPF patients show a progressive decline in pulmonary function leading to respiratory failure, which is caused by excessive extracellular matrix (ECM) deposition in the lungs. The pathophysiology of IPF is complex and although numerous molecular pathways and cell populations have been shown to drive disease progression, its etiology remains only partly understood [6, 7]. Better understanding of disease driving pathways and cell populations involved might lead to novel treatment options that are able to stop or reverse disease progression.

A specific cell population implicated in the development and progression of IPF are macrophages. Macrophages are highly plastic and can polarize toward different activation states in response to internal or external cues [8]. Lung macrophages are the major immune sentinels in steady state lung tissue and play a crucial role in maintaining lung homeostasis [9]. Of note however, macrophages are also crucial regulators of the development of lung fibrosis [10, 11] and it is well recognized that macrophages play a dual role in pulmonary fibrosis. They can promote fibrosis by secreting pro-fibrotic soluble mediators, chemokines, and matrix metalloproteases. On the other hand, macrophages can enhance the resolution of fibrosis by stimulating ECM processing through secretion of matrix metalloproteases and/or promoting epithelial regeneration [10, 12]. Therefore, the biology of macrophages in pulmonary fibrosis should be tightly regulated and abnormal macrophage polarization has been suggested to contribute to this lung disease [12]. The mechanisms underlying macrophage biology in pulmonary fibrosis remain poorly understood however.

Epigenetic regulation has been increasingly recognized to play a crucial role in various biological processes and diseases. Alternation of DNA methylation, one of the most intensively studied epigenetic regulations of gene expression, was documented in the lungs of IPF patients and DNA methylation levels were negatively associated with related gene expression [13, 14]. DNA methylation is established and maintained by DNA methyltransferases (DNMTs) [15], whose expression has been reported to be altered in IPF [13]. There are three well documented DNMTs, DNMT1, DNMT3A and DNMT3B where DNMT1 is mainly involved in the maintenance of DNA methylation, whereas the latter two are important for de novo DNA methylation [16]. DNMT3B is the major DNMT for de novo DNA methylation and has been reported to regulate macrophage polarization [17].

Therefore, given the importance of macrophages in the development of pulmonary fibrosis [23] and the role of DNMT3B in macrophage polarization [17], we hypothesized that macrophage specific DNMT3B may play a role in fibrosis development. To test this assumption, we assessed macrophage subpopulations and fibrotic responses in the lungs of myeloid cell specific *Dnmt3b* deficient mice during bleomycin-induced pulmonary fibrosis. The results demonstrate that myeloid DNMT3B limits the development of pulmonary fibrosis by limiting M2 macrophage polarization potentially by methylation of the promoter of the gene encoding *arginase 1* (*Arg1*).

## Materials and methods

### Mice

Homozygous *Dnmt3b*^*fl/fl*^ mice (RBRC03733, RIKEN BRC, Tsukuba, Japan) [19] were crossed with *LysM*^*cre*^ mice (Jackson Laboratory, Bar Harbor, ME) [20] to generate myeloid cell specific Dnmt3b deficient (*Dnmt3b*^*fl/fl*^*LysM*^*Cre*^) mice. Dnmt3b deficiency in macrophages of *Dnmt3b*^*fl/fl*^*LysM*^*Cre*^ mice was confirmed as previously described [21]. Animals were maintained at the animal facility of the Academic Medical Center (University of Amsterdam) with free access to food and water. All mice experiments were approved by the Animal Care and Use Committee of the Academic Medical Center.

### Bone marrow derived macrophages (BMDMs)

BMDMs were generated as previously described [22]. Briefly, femur and tibia were obtained from 8–12 weeks old *Dnmt3b*^*fl/fl*^*LysM*^*Cre*^ mice and littermate *Dnmt3b*^*fl/fl*^ mice. Bone marrow was flushed out of the femur and tibia with medium and cells were resuspended and plated. Bone marrow cells were subsequently incubated for 7 days in RPMI 1640 medium supplemented with 10% FCS, 1% penicillin/streptomycin and 15% L929 mouse fibroblast supernatant in order to differentiate the cells into BMDMs. Subsequently, the cells were plated in a 24-well plate in RPMI 1640 medium supplemented with 10% FCS and 1% penicillin/streptomycin and incubated overnight. Cells were stimulated with recombinant murine interleukin (IL)4 (PeproTech EC, London, UK) at a final concentration of 20 ng/ml, recombinant human transforming growth factor-beta (TGFB1, Tebu-bio, Offenbach, Germany) at a final concentration of 5 ng/ml, or lipopolysaccharides (LPS, from E. coli O111:B4; InvivoGen, Toulouse, France) at a final concentration of 100 ng/ml for 2, 6, 12 and 24 h. Supernatants were collected and stored at − 20 °C for further analysis and cells were collected and stored at − 80 °C in RNA isolation buffer (Nucleospin RNA, Machery-Nagel, Leiden, Netherlands) or DNA isolation buffer (DNeasy Blood and Tissue Kit, Qiagen, Hilden, Germany).

### Quantitative reverse transcription PCR (RT-qPCR)

Total RNA from BMDMs, broncho-alveolar lavage fluid (BALF) cells and lung homogenates was isolated using NucleoSpin RNA columns (Bioke, Leiden, Netherlands) according to the manufacturer’s recommendations. All RNA samples were quantified by spectrophotometry and stored at − 80 °C until further analysis. cDNA was prepared using the M-MLV Reverse Transcriptase kit (Promega, Leiden, Netherlands) according to manufacturer’s instructions. Gene expression analysis was performed using a Roche LightCycler 480 thermocycler with SensiFAST Real-time PCR kit (Bioline, London, UK) using the gene specific primers listed in Table 1. Gene specific expression levels were normalized to expression levels of the gene encoding *hypoxanthine–guanine phosphoribosyltransferase* (*Hprt*).

**Table 1:** Primer sequences used for RT-qPCR

Specie	Gene	Forward	Reverse
Mouse	*Arg1*	CTGGGAATCTGCATGGGCAA	GTCTACGTCTCGCAAGCCAA
	*Fizz1*	CGTGGAGAATAAGGTCAAGGAAC	CACAAGCACACCCAGTAGCAG
	*Spp1*	GCCGAGGTGATAGCTTGGCTTATG	CTCTCCTGGCTCTCTTTGGAATGC
	*Pdgfα*	CCAACCTGAACCCAGACCAT	CAAAGACCGCACGCACATT
	*Mmp8*	TTGCCCATGCCTTTCAACCA	TGAGCAGCCACGAGAAATAGG
	*Mmp12*	GGAACTTGCAGTCGGAGGGA	AATTCACTGTCATTCATGGGAGCA
	*Fn1*	AGAGGAGGCACAAGGTTCGG	GACAACCGCTCCCACTCCTC
	*Il-6*	CTTCCTACCCCAATTTCCAATGCT	TCTTGGTCCTTAGCCACTCCTT
	*Tnf*	CGAGTGACAAGCCTGTAGCC	CCTTGAAGAGAACCTGGGAGT
	*Hprt*	AGTCAAGGGCATATCCAACA	CAAACTTTGCTTTCCGGGT

### Methylated DNA immunoprecipitation (MeDIP)

MeDIP analysis was performed as previously described [23]. In brief, DNA from BMDMs was isolated using the DNeasy Blood and Tissue Kit (Qiagen 174 GmbH, Hilden, Germany) according to the manufacturer’s instructions. Subsequently, DNA was sonicated into 200 to 1000 bp DNA fragments. Methylated DNA fragments were then immunoprecipitated using the Methylamp Methylated DNA Capture Kit (Epigentek, Farmingdale, NY) according to the manufacturer’s instructions. DNA methylation levels at two regions (P1 and P2) of the Arg1 promoter were measured by qPCR with two pairs of primers (P1 Forward: 5-TTCCTCTGATGGGGAGGTTCT-3′, P1 Reverse 5′-CCCTAAAAGACAGAGGGCACA-3′; P2 Forward: 5-TGAACAGGCTGTATTAGCCAACA-3′, P2 Reverse 5′-AGCACCCTCAACCCAAAGTG-3’).

### Animal model of pulmonary fibrosis

Pulmonary fibrosis was induced by a single intranasal dose of bleomycin (Sigma, St-Louis, MO) at 2 U/kg body weight as described before [24]. All mice were euthanized by heart puncture after injection of ketamine/medotomidine at 21 days after bleomycin installation. A bronchoalveolar lavage (BAL) was performed by flushing the right lung with 2 aliquots of 0.5 ml sterile phosphate-buffered saline after ligation of the left tracheal bronchus after which the lung was collected and homogenized as described before [25] and stored at − 20 °C until further analysis. The left lung was preserved in 10% formalin for 24 h and used for histopathology. BAL fluid was centrifuged at 800 g for 10 min at 4 °C after which the supernatant was stored at − 20 °C until further analysis. BALF cells were counted using a hemocytometer (Beckman Coulter, Fullerton, CA) and differential cell populations were subsequently determined by flow cytometry (see below). BALF cells were lysed in RNA isolation lysis buffer. BALF supernatants and lung homogenates were stored at − 20 °C until further analysis.

### Hydroxyproline assay

Hydroxyproline levels were measured in lung homogenates using a hydroxyproline assay kit (Sigma, St-Louis, MO) according to the manufacturer’s instructions and as described before [24].

### Enzyme-linked immunosorbent assays (ELISA)

Mouse Monocyte Chemoattractant Protein 1 (MCP1) and active TGFB1 levels were measured in BALF and lung homogenates, and IL6 and Tumor Necrosis Factor (TNF)A levels were measured in cell culture supernatants by specific ELISAs (all R&D Systems, Minneapolis, MN) according to manufacturer’s instructions.

### Flow cytometry

Differential cell counts in BALF were determined by flow cytometry as described [26, 27]. Briefly, BALF cells were resuspended in FACS buffer (5% BSA, 0.35 mM EDTA, 0.01% NaN3). Cell staining was performed according to manufacturer’s recommendations using fixable viability dye eFluor 780, rat anti mouse-CD16/CD32 (clone 93), rat anti mouse-CD45 PE-eFluor610 (30-F11), rat anti-mouse CD11b PE-Cy7 (clone M1/70), rat anti-mouse Siglec-F Alexa Fluor 647 (clone E50-2440) (all from BD Biosciences, San Jose, CA); anti-mouse CD64 PerCP-Cy5-5 (clone X54-5/7.1) and rat anti-mouse Ly-6G FITC (clone 1A8) (both from Biolegend, San Diego, CA). Flow cytometry analysis was performed using a FACSCANTO II (Becton Dickinson, Franklin Lakes, NJ) and data were analyzed using FlowJo software (Becton Dickinson). The gating strategy that was used to analyze the different populations is shown in supplementary Figure S1. Fibrotic macrophage population was defined as CD45 + CD64 + SigecF^low^CD11b^hi^; classical alveolar macrophages were defined as CD45 + CD64 + SiglecF^hi^CD11b^low^.

### Western blot

Fibronectin (FN1), collagen type I (COL1A1) and α-tubulin protein levels were measured in lung homogenates by western blot as described [25]. Western blot results were quantified using ImageJ (National Institutes of Health and the Laboratory for Optical and Computational Instrumentation, University of Wisconsin, Madison, WI).

### Histological analysis

Histological examination was performed essentially as described before [24, 28]. After the left lungs were embedded in paraffin, lung sections were prepared and stained with hematoxylin and eosin (H&E) according to routine procedures. The severity of fibrosis was evaluated according to the Ashcroft scoring system [29] by two independent observers in a blinded fashion. An average of 10 fields for each lung section were selected and scored. The results were reported as the average score of the individual field scores.

### Statistics

Statistical analyses were conducted using GraphPad Prism 8 (GraphPad software, San Diego, CA). Comparisons between two conditions were analyzed using Mann–Whitney analysis. P values of less than 0.05 were considered significant.

## Results

### DNMT3B deficiency promotes alternative macrophage polarization in vitro

**Fig. 1:**
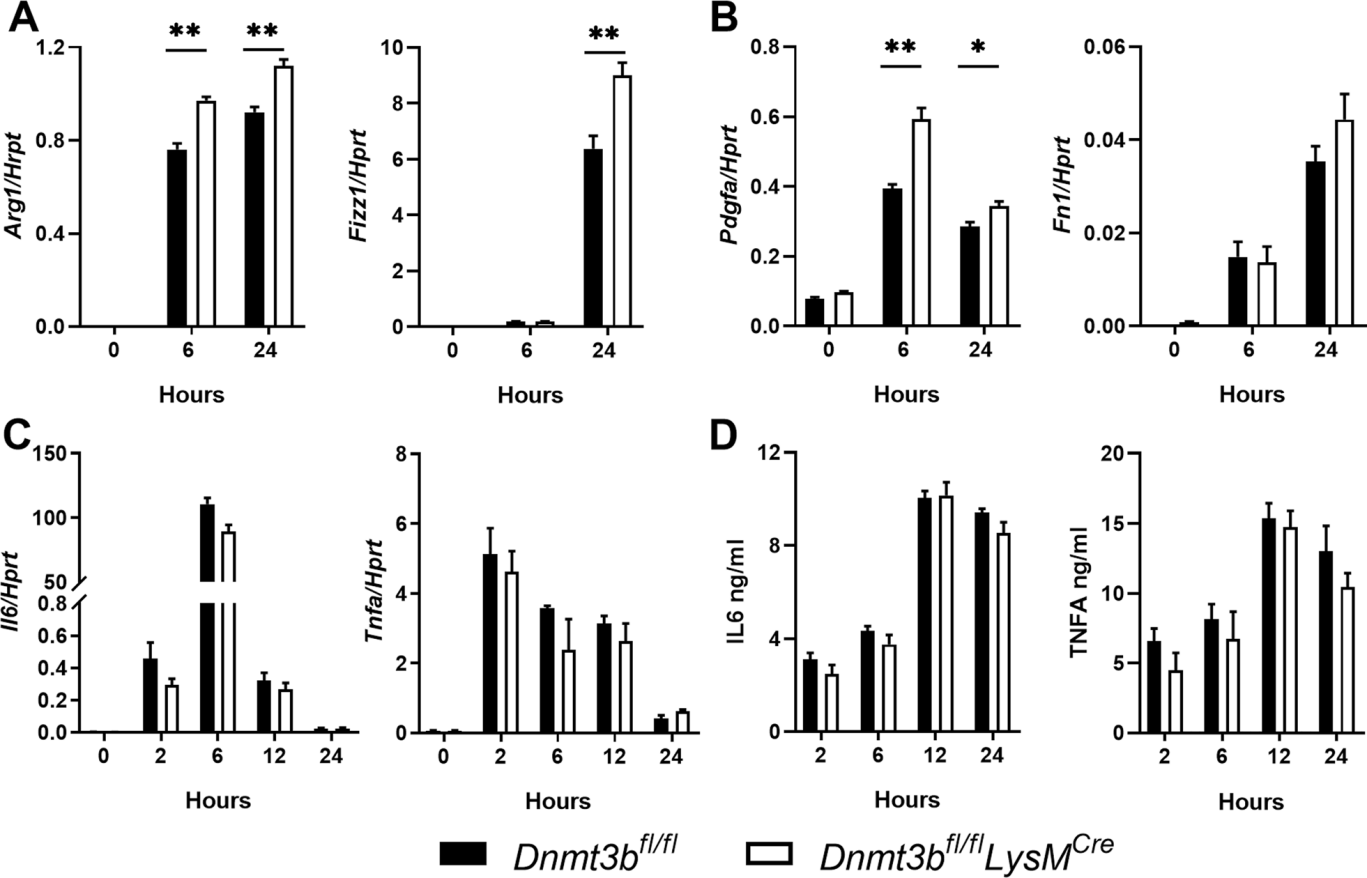
Myeloid Dnmt3b deficiency promotes alternative polarization of bone marrow-derived macrophages (BMDMs). **A** Relative gene expression of *Arg1* (Arginase 1) and *Fizz1* (Resistin like alpha) in BMDMs of control (*Dnmt3b*^*fl/fl*^) and DNMT3B conditional knockout (*Dnmt3b*^*fl/fl*^*LysM*^*cre*^) mice stimulated with IL4 (20 ng/ml) for 0, 6 and 24 h determined by RT-qPCR. Expression levels are relative to *Hprt* (hypoxanthine–guanine phosphoribosyltransferase). **B** Relative gene expression of *Pdgfa* (platelet derived growth factor subunit A) and *Fn1* (Fibronectin) in BMDMs of control (*Dnmt3b*^*fl/fl*^) and DNMT3B conditional knockout (*Dnmt3b*^*fl/fl*^*LysM*^*cre*^) mice stimulated with TGFB1 (5 ng/ml) for 0, 6 and 24 h determined by RT-qPCR. Expression levels are relative to *Hprt*. **C** Relative gene expression of *Il6* (Interleukin 6) and *Tnfa* (Tumor necrosis factor-α) in BMDMs of control (*Dnmt3b*^*fl/fl*^) and DNMT3B conditional knockout (*Dnmt3b*^*fl/fl*^*LysM*^*cre*^) mice stimulated with lipopolysaccharide (LPS, 100 ng/ml) for 0, 2, 6, 12 and 24 h determined by RT-qPCR. Expression levels are relative to *Hprt*. **D** IL6 and TNFA protein levels in the supernatant of BMDMs from control (*Dnmt3b*^*fl/fl*^) and DNMT3B conditional knockout (*Dnmt3b*^*fl/fl*^*LysM*^*cre*^) mice stimulated with LPS (100 ng/ml) for 0, 2, 6, 12 and 24 h determined by ELISA. Data represent results one of three independent experiments showing similar results. Data are presented as mean ± SEM, n = 6 per group; Black bars: Littermate control mice, open bars: myeloid specific DNMT3B deficient mice. *p < 0.05, **p < 0.01

Polarization of macrophages towards a M2 type has been implicated in the pathogenesis of lung fibrosis [12]. To determine whether DNMT3B affects macrophage polarization in vitro we stimulated BMDMs from *Dnmt3b*^*fl/fl*^*LysM*^*Cre*^ and *Dnmt3b*^*fl/fl*^ control mice with IL4, TGFB1 or LPS. IL4 is a strong inducer of alternative macrophage polarization that can be recognized by high gene expression of *Arg1* and *Fizz1* [30]. Both *Arg1* and *Fizz1* mRNA levels were increased at 6 and 24 h in BMDMs stimulated with IL4, which was further enhanced by DNMT3B deficiency (Fig. 1A). Also TGFB1 induces alternative macrophage polarization, which is accompanied by extensive expression of profibrotic genes *Fn1* and *Pdgfa* [31]. mRNA levels of *Fn1* and *Pdgfa* were increased upon TGFB1 stimulation, which was further increased in DNMT3B deficient macrophages, although for *Fn1* the difference between genotypes did not reach statistical significance (Fig. 1B). These results suggest that DNMT3B limits alternative macrophage polarization, corroborating previous reports [17, 32]. Next to alternative macrophages, classically activated macrophages are present in the lungs of IPF patients [33]. To examine whether DNMT3B also regulates inflammatory/classic macrophage activation, BMDMs were stimulated with LPS for 2, 6, 12 and 24 h. The mRNA expression of the pro-inflammatory genes *Il6* and *Tnfa* and their corresponding proteins was strongly induced by LPS stimulation, but both mRNA and protein levels of these mediators were comparable in DNMT3B deficient and control macrophages (Fig. 1C, D). Collectively, these findings suggest that DNMT3B represses alternative macrophage polarization but does not regulate classical macrophage polarization in vitro.

### Myeloid DNMT3B inhibits alternative macrophage polarization during bleomycin-induced pulmonary fibrosis

**Fig. 2:**
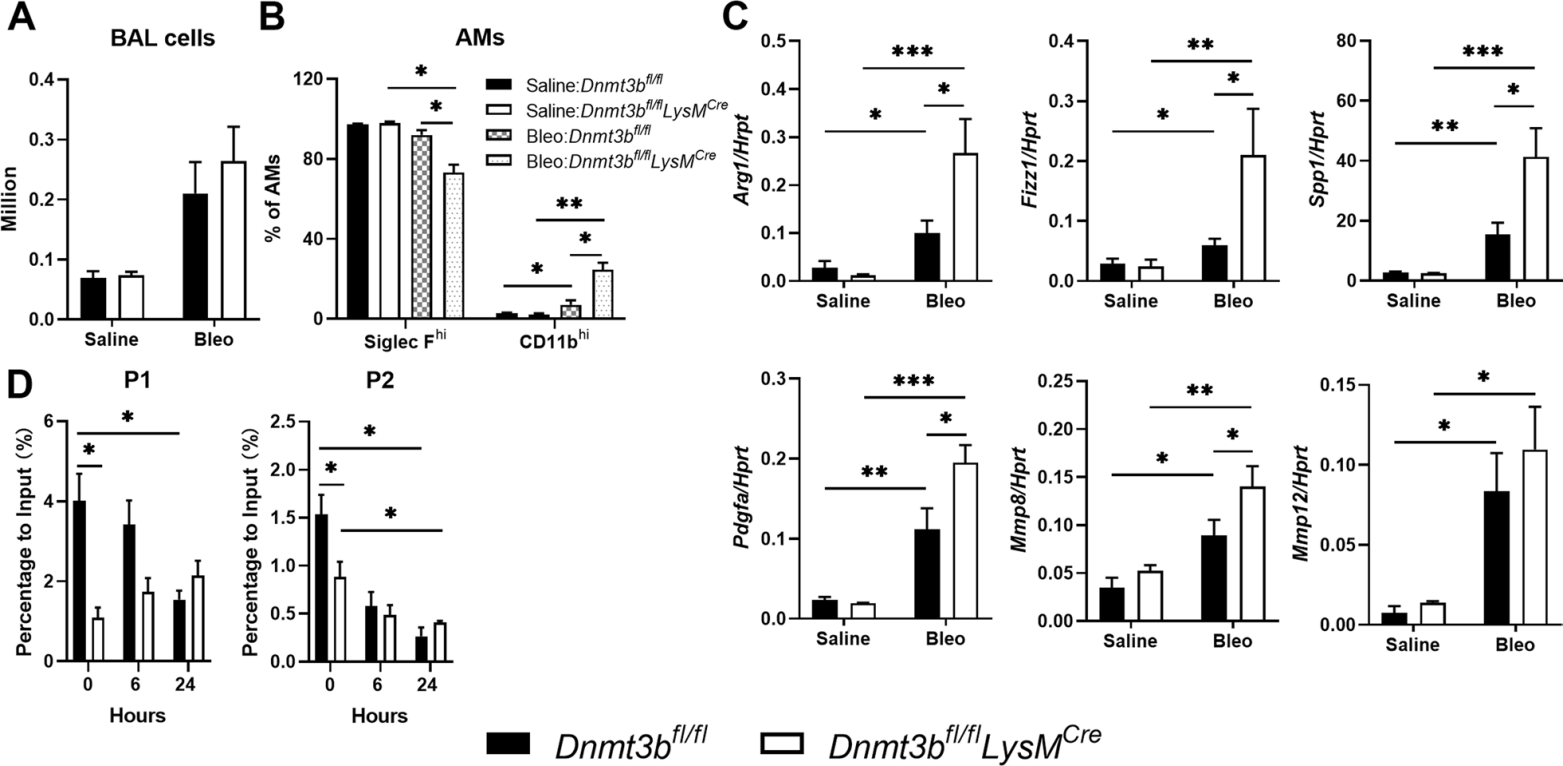
Myeloid DNMT3B inhibits alternative macrophage polarization in the lungs during bleomycin-induced pulmonary fibrosis. **A** Total cell numbers in bronchoalveolar lavage fluid (BALF) of control (*Dnmt3b*^*fl/fl*^) and DNMT3B conditional knockout (*Dnmt3b*^*fl/fl*^*LysM*^*cre*^) mice 21 days after saline of bleomycin treatment. **B** Percentage of classical alveolar macrophages (Siglec F^hi^ AMs) and alternative alveolar macrophage populations (CD11b^hi^ AMs) in BALF of control (*Dnmt3b*^*fl/fl*^) and DNMT3B conditional knockout (*Dnmt3b*^*fl/fl*^*LysM*^*cre*^) mice 21 days after saline or bleomycin treatment analyzed by flow cytometry. **C** Relative gene expression of alternative macrophage markers *Arg1* (Arginase 1), *Fizz1* (Resistin like alpha), *Spp1* (Secreted Phosphoprotein 1), *Pdgfa* (platelet derived growth factor subunit A), *Mmp8* (matrix metalloproteinase 8) and *Mmp12* (matrix metalloproteinase 12) in BALF cells of control (*Dnmt3b*^*fl/fl*^) and DNMT3B conditional knockout (*Dnmt3b*^*fl/fl*^*LysM*^*cre*^) mice 21 days after saline or bleomycin treatment determined by RT-qPCR. Expression levels are relative to *Hprt* (hypoxanthine–guanine phosphoribosyltransferase). **D** Percentage of DNA methylation at region p1 and p2 of the *Arg1* promoter in control (*Dnmt3b*^*fl/fl*^) and DNMT3B conditional knockout (*Dnmt3b*^*fl/fl*^*LysM*^*cre*^) bone marrow-derived macrophages (BMDMs) 0, 6 and 24 h after IL4 (20 ng/ml) stimulation. Data are presented as mean ± SEM, n = 10 mice per group (bleomycin treated) or 4 mice per group (saline control) in **A**, **B**; n = 6 in C. Black bars: littermate control mice, open bars: myeloid specific DNMT3B deficient mice. *p < 0.05, **p < 0.01, ***p < 0.001

To determine the potential role of DNMT3B in macrophage polarization during pulmonary fibrosis in vivo, we subjected myeloid cell specific DNMT3B deficient mice (*Dnmt3b*^*fl/fl*^*LysM*^*Cre*^ mice) and their littermate controls (*Dnmt3b*^*fl/fl*^ mice) to the bleomycin-induced lung fibrosis model. Monocyte derived macrophages are reported to be the major source of the macrophage population that contributes to the development of pulmonary fibrosis [26, 34] and therefore we first assessed cell influx into the lung by counting the total cell number in BALF. As shown in Fig. 2A, the total cell number in BALF was increased 21 days after bleomycin instillation to a similar level in myeloid DNMT3B deficient mice and control mice. To further substantiate the alternative macrophage population in fibrotic lungs we quantified the expression of CD11b, a marker for alternative macrophages contributing to pulmonary fibrosis [35], and Siglec F in BAL cells. The fibrotic macrophage population (SiglecF^low^CD11b^hi^) was increased in both control and DNMT3B conditional knockout mice after bleomycin treatment, and this macrophage population was significantly larger in DNMT3B conditional knockout mice when compared to control littermates (Fig. 2B). On the other hand, the classic alveolar macrophage population (SiglecF^hi^CD11b^low^) were significantly lower in myeloid DNMT3B deficient mice compared to control mice (Fig. 2B). BALF and lung tissue levels of MCP1, the most important chemotactic protein for monocyte migration [36], were increased upon bleomycin treatment but not affected by myeloid cell DNMT3B deficiency (Additional file 1: Figure S2A). To further substantiate the impact of myeloid DNMT3B on the alternative macrophage population, gene expression of *Arg1, Fizz1, Spp1, Pdgfa*, *Mmp8* and *Mmp12* were analyzed in BALF cells; the expression of these alternative macrophage markers was increased after bleomycin treatment, which was significantly enhanced in myeloid DNMT3B deficient mice, except for *Mmp12* (Fig. 2C). On the contrary, gene expression levels of classical macrophage markers *Il6* and *Tnfa* were not affected by bleomycin treatment or DNMT3B deficiency (Additional file 1: Figure S2B). As a de novo DNA methyltransferase, the most well-recognized mechanism underlying DNMT3B mediated gene expression is DNA methylation [37]. We therefore examined DNA methylation levels at the promoter regions of *Arg1* in BMDMs with and without IL4 stimulation in vitro. As shown in Fig. 2D, DNA methylation within the Arg1 promoter was significantly higher in unstimulated BMDMs at both regions analyzed. Methylation of the *Arg1* promoter was rapidly decreased upon IL4 stimulation in control BMDMs, whereas methylation was unaltered in DNMT3B deficient BMDMs. These results suggest that DNMT3B regulates alternative macrophage polarization at least in part by mediating DNA methylation of the *Arg1* promoter.

### Myeloid Dnmt3b deficiency exacerbates bleomycin-induced pulmonary fibrosis

**Fig. 3:**
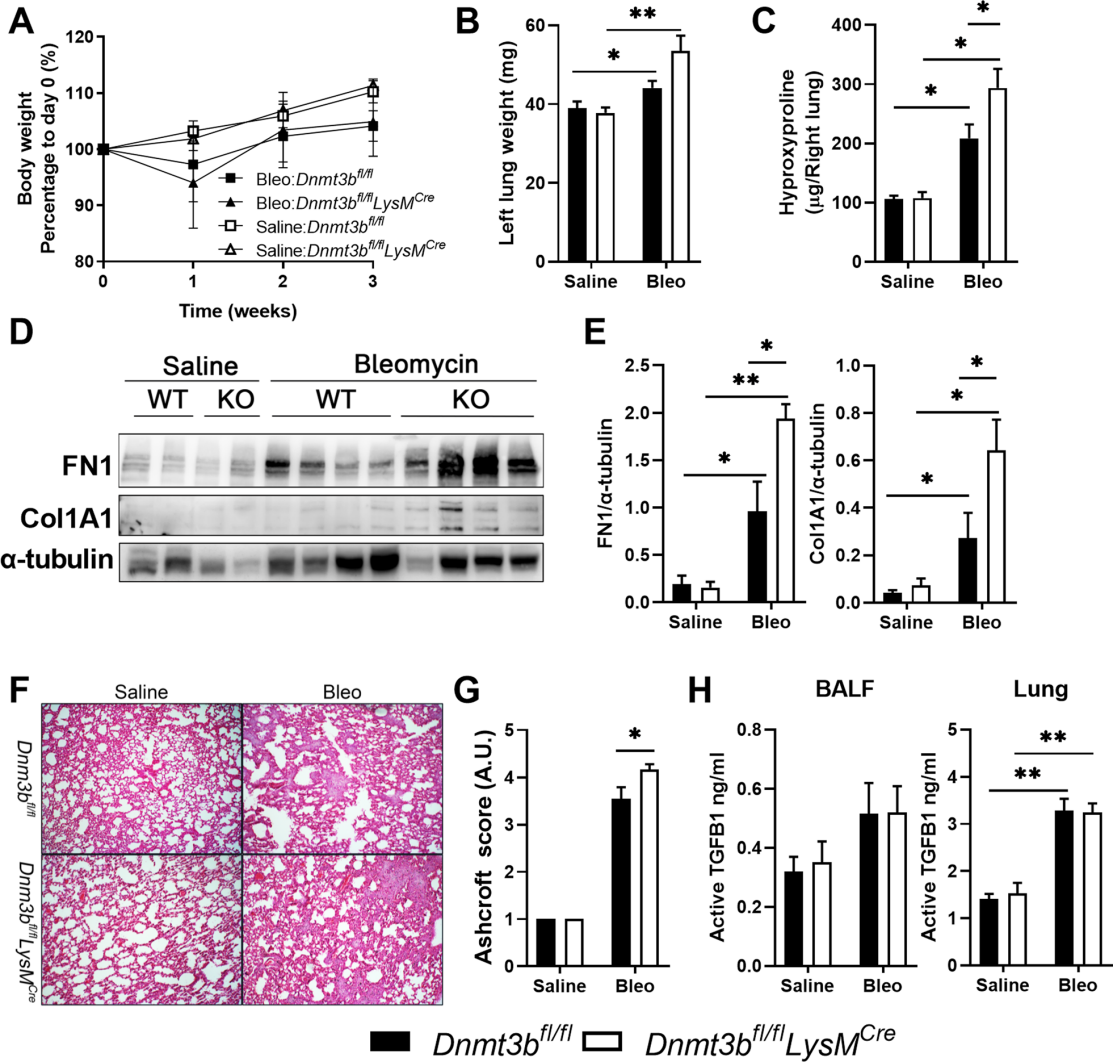
Myeloid DNMT3B deficiency promotes bleomycin induced pulmonary fibrosis. **A** Body weight of control (*Dnmt3b*^*fl/fl*^) and DNMT3B conditional knockout (*Dnmt3b*^*fl/fl*^*LysM*^*cre*^) mice over time after bleomycin or saline treatment. **B** Lung weight of left lung lobes of control (*Dnmt3b*^*fl/fl*^) and DNMT3B conditional knockout (*Dnmt3b*^*fl/fl*^*LysM*^*cre*^) mice 21 days after bleomycin (Bleo) or saline treatment. **C** Collagen expression as measured by hydroxyproline levels in the right lung of control (*Dnmt3b*^*fl/fl*^) and DNMT3B conditional knockout (*Dnmt3b*^*fl/fl*^*LysM*^*cre*^) mice 21 days after bleomycin (Bleo) or saline treatment. **D** Representative pictures of western blot assays for the detection of Fibronectin (FN1) and Collagen type I (COL1A1) protein levels in lung homogenates of control (ctrl) and DNMT3B conditional knockout (KO) mice 21 days after bleomycin treatment. α-Tubulin serves as a loading control. **E** Quantification of FN1 and COL1A1 expression relative to α-tubulin levels of Western blots depicted in **D**. **F** Representative H&E-stained lung tissue sections of control (*Dnmt3b*^*fl/fl*^) and DNMT3B conditional knockout mice (*Dnmt3b*^*fl/fl*^*LysM*^*cre*^) 21 days after saline or bleomycin treatment (20x). **G** Quantification of pulmonary fibrosis using the Ashcroft score in control (*Dnmt3b*^*fl/fl*^) and DNMT3B conditional knockout (*Dnmt3b*^*fl/fl*^*LysM*^*cre*^) mice 21 days after saline or bleomycin treatment. **H** Active TGFB1 protein levels in bronchoalveolar lavage fluid (BALF) and lung homogenates of control (*Dnmt3b*^*fl/fl*^) and DNMT3B conditional knockout (*Dnmt3b*^*fl/fl*^*LysM*^*cre*^) mice 21 days after saline or bleomycin treatment determined by ELISA. Data are presented as mean ± SEM, n = 10 mice per group (bleomycin treated) or 4 mice per group (saline control). Bleo: bleomycin; Black bars: littermate control mice, open bars: myeloid specific DNMT3B deficient mice. *p < 0.05, **p < 0.01

Alternatively activated macrophages have been suggested to promote the development of bleomycin induced fibrosis [26, 34, 35] and therefore the effect of DNMT3B on macrophage polarization may affect the development of pulmonary fibrosis. In order to determine this potential effect, fibrotic parameters were assessed 21 days after bleomycin treatment, i.e., typically when the peak of fibrosis is observed [38]. Bleomycin administration led to transient body weight loss which was not affected by myeloid DNMT3B deficiency (Fig. 3A). Lung weight was increased upon bleomycin treatment which was significantly enhanced in DNMT3B conditional knockout mice (Fig. 3B). Lung levels of hydroxyproline, a marker for collagen content, were increased upon bleomycin treatment in both mouse strains, but significantly higher in the lungs of DNMT3B conditional knockout than in control mice (Fig. 3C). In line, fibronectin and collagen type I protein levels in the lungs were increased upon bleomycin administration and these levels were higher in DNMT3B conditional knockout mice (Fig. 3D, E). Histological examination of the lungs showed extensive patchy areas of fibrosis at day 21 (Fig. 3F) which were significantly more present in DNMT3B conditional knockout mice, as indicated by quantification using the Ashcroft scoring system (Fig. 3G). TGFB1 is an important cytokine driving pulmonary fibrosis and mostly produced by macrophages during fibrosis [39]. As shown in Fig. 3H, active TGFB1 levels in both BALF and lung homogenates were increased upon bleomycin administration to a similar extent in myeloid DNMT3B deficient mice and control littermates. Collectively, myeloid cell specific DNMT3B deficiency exacerbates the development of pulmonary fibrosis induced by bleomycin potentially by regulating alternative macrophage polarization.

## Discussion

IPF is a complex lung disease believed to result from an atypical response to injury of the epithelium that leads to an aberrant wound healing response and the deposition of extracellular matrix [40]. Macrophages play crucial roles in this process and their activation is tightly regulated during IPF. Here we show that the loss of DNMT3B in macrophages promotes alternative, but not classic, macrophage polarization, and is associated with enhanced bleomycin-induced pulmonary fibrosis.

Macrophages are highly heterogeneous and have many faces in the fibrotic lung [41, 42]. Macrophages are highly plastic, and their polarization is determined by the local environment [9, 43]. Both classical (M1) and alternative (M2) macrophage subpopulations have been found in IPF lungs, where they seem to play dual roles in the development of pulmonary fibrosis [12]. CD11b^hi^ alternative macrophages promote the development of bleomycin-induced fibrosis as elegantly shown by the fact that depletion of this specific subset prevents fibrosis [44]. In line, we here show that the number of CD11b^hi^ alternative macrophages in the alveolar space was significantly increased upon bleomycin administration which was accompanied by increased expression of profibrotic macrophage markers in these cells. Interestingly, both the number of CD11b^hi^ macrophages and the expression of profibrotic markers were further increased in macrophages of myeloid DNMT3B deficient mice challenged with bleomycin compared to control challenged mice. The increased alternative macrophage polarization in myeloid DNMT3B deficient mice was accompanied by increased pulmonary fibrosis which suggests that macrophage DNMT3B inhibits the development of pulmonary fibrosis by restricting alternative macrophage polarization.

It has been shown that recruitment of monocytes contributes to macrophage populations during the development of pulmonary fibrosis [26, 34]. Therefore, we assessed whether macrophage recruitment was affected by myeloid DNMT3B. However, we did not observe a difference in cell numbers in BALF nor in MCP1 levels, the major monocyte chemoattractant, between control and myeloid DNMT3B deficient mice. It seems therefore unlikely that myeloid DNMT3B is involved in the recruitment of macrophages; rather its role seems restricted to alternative macrophage polarization. Of interest, a very recent study showed that the monocyte-derived macrophage population is not altered in IPF lungs compared to normal lungs, suggesting that the role of monocyte-derived macrophages in the development of pulmonary fibrosis is limited in human disease [45]. The same study that precludes monocyte-derived macrophages as key mediators in IPF did however show that two specific resident-like macrophage populations are increased in IPF [45]. One of these resident-like macrophage subsets, i.e. the SPP1^hi^/MERTK^hi^ population, may be particularly interesting as we here also show that SPP1 expression was strongly increased in BALF cells upon bleomycin treatment which was even further elevated in myeloid DNMT3B deficient mice, suggesting that these results may be relevant for the human situation.

Typically, de novo DNA methyltransferases regulate gene expression by promoter methylation of target genes [16]. This prompted us to investigate whether *Arg1* promoter methylation was altered in DNMT3B deficient BMDMs. Interestingly, promoter methylation of *Arg1* was higher in DNMT3b positive BMDMs compared to DNMT3B deficient BMDMs suggesting that *Arg1* is a direct target of DNMT3B and already at baseline DNMT3B inhibits *Arg1* expression by methylation of its promotor. This is in line with a previous study showing that the expression of *Arg1* is negatively regulated by DNA methylation in BMDMs [46]. DNA methylation in lung tissues of IPF patients was altered compared to healthy controls suggesting that such epigenetic modifications may play a role in the pathophysiology of the disease [13, 14]. This is in line with a recent study showing that aberrant DNA methylation in alveolar macrophages is associated with macrophage differentiation and is associated with IPF pathogenesis [47]. Future studies focusing on the identification of other DNMT3B specific targets for DNA methylation will need to be employed and also DNA methylation levels within the promotor regions of DNMT3B specific targets in macrophages obtained from IPF patients need to be assessed to unveil the role of DNMT3B in pulmonary fibrosis. Together with DNMT3B, DNMT3A also catalyzes de novo DNA methylation, while the DNMT1 maintains DNA methylation [16]. Therefore, we cannot rule out a potential role of DNMT3A and DNMT1 in regulating macrophage polarization during fibrosis, and other studies have already shown that both DNMT1 and DNMT3A play a role in the development of pulmonary fibrosis by modulating DNA methylation in lung fibroblasts and alveolar epithelial cells [18].

Notably, classic macrophage polarization was not affected by DNMT3B deletion, whereas others have shown that DNMT3B can promote classical macrophage polarization in RAW264.7 macrophages [17]. The fact that we did not observe such an effect upon LPS stimulation of BMDMs may be explained by the difference in cell type that was used. Moreover, the reason that we did not find a difference in classical macrophage polarization upon bleomycin administration may be explained by the time point that was analyzed. Indeed, 21 days post bleomycin instillation is typically the peak of the fibrotic stage whereas the inflammatory stage peaks around day 3 [48, 49]. Most importantly, in the aforementioned study DNMT3B did inhibit alternative macrophage polarization similarly to our resultsas shown by knockdown and overexpression experiments of DNMT3B using RAW264.7 macrophages [17] which underscores the role of DNMT3B in alternative macrophage polarization in vitro. Here we show that this mechanism is also relevant in the setting of experimental pulmonary fibrosis in vivo.

## Conclusions

Overall, the present study demonstrates that myeloid DNMT3B represses fibrotic macrophage polarization and ameliorates the development of bleomycin induced pulmonary fibrosis.

## Supplementary Information

**Additional file 1.** Supplementary materials.

## Data Availability

All data generated and analyzed during the study are included in the published article and can be shared upon request.

## Abbreviations

IPF: Idiopathic pulmonary fibrosis
Dnmt: DNA methyltransferase
TGFB1: Transforming growth factor β1
IL: Interleukin
ECM: Extracellular matrix
Hprt: Hypoxanthine–guanine phosphoribosyltransferase
MeDIP: Methylated DNA immunoprecipitation
BAL: Bronchoalveolar lavage
MCP1: Monocyte chemoattractant Protein 1
TNFA: Tumor Necrosis Factor α
H&E: Hematoxylin–eosin staining
BMDM: Bone-marrow-derived macrophage
LPS: Lipopolysaccharide
