# Supplementary material for: Myeloid DNA methyltransferase3b deficiency aggravates pulmonary fibrosis by enhancing profibrotic macrophage activation
Source: Respir Res. 2022 Jun 20;23:162. doi: 10.1186/s12931-022-02088-5 (PMC9210707; doi:10.1186/s12931-022-02088-5)
Supplement: Supplementary file 1 — Additional file 1. Supplementary materials. [file 12931_2022_2088_MOESM1_ESM.docx]

**Supplementary materials**

**Myeloid DNA methyltransferase3b deficiency aggravates pulmonary fibrosis by enhancing profibrotic macrophage activation**

Wanhai Qin^1^, C. Arnold Spek^1^, Brendon P. Scicluna^1,2^, Tom van der Poll^1,3^, JanWillem Duitman^1,4^

Amsterdam University Medical Centers, location Academic Medical Center, University of Amsterdam, Amsterdam, the Netherlands: ^1^Center of Experimental and Molecular Medicine, ^2^Department of Clinical Epidemiology, Biostatistics, and Bioinformatics, ^3^Division of Infectious Diseases. ^4^Department of Pulmonary Medicine.

**Supplementary figure 1**

**
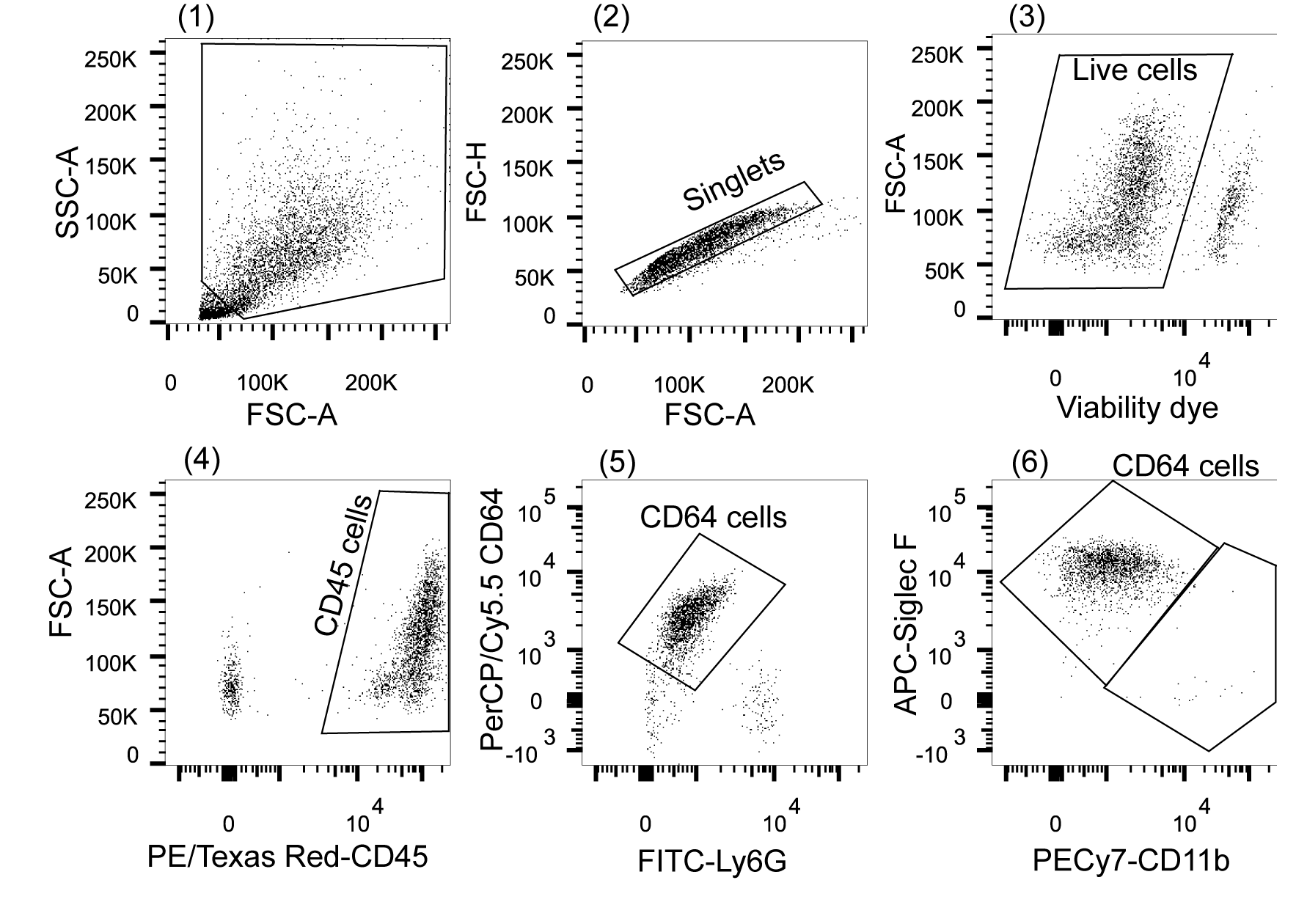
**

**Supplementary figure 1. Flow cytometry gating strategy to determine the percentage of macrophage subsets in bronchoalveolar lavage fluid.** The percentage of classic alveolar macrophages (CD45+CD64+Siglec F^hi^CD11b^low^) and profibrotic macrophages (CD45+CD64+Siglec F^low^CD11b^hi^) were determined in the live and single cell population as depicted.

**Supplementary figure 2**

**
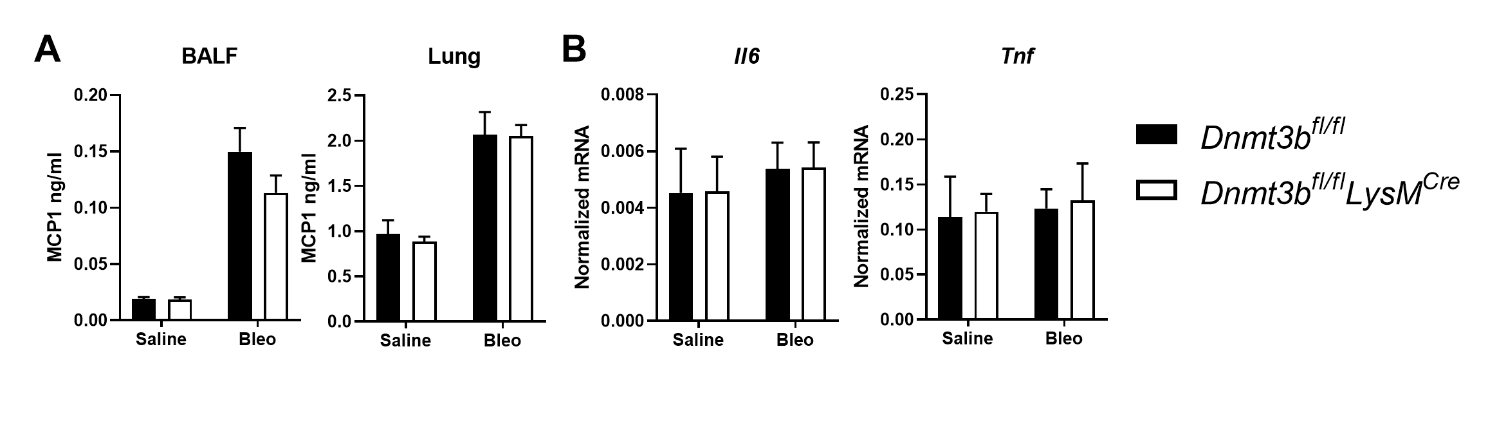
**

**Supplementary figure 2. Myeloid Dnmt3b does not affect monocyte migration or classic macrophage polarization. (A)** MCP-1 protein levels in bronchoalveolar lavage fluid (BALF) and lung homogenates (Lung) of control (*Dnmt3b^fl/fl^*) and Dnmt3b conditional knockout (*Dnmt3b^fl/fl^LysM^cre^*) mice 21 days after saline or bleomycin treatment determined by ELISA. **(B)** Relative gene expression of and classic macrophage markers Il-6 (Interleukin 6) and Tnf (Tumor necrosis factor) in BALF cells of control (*Dnmt3b^fl/fl^*) and Dnmt3b conditional knockout (*Dnmt3b^fl/fl^LysM^cre^*) mice 21 days after saline or bleomycin treatment determined by RT-qPCR. Expression levels are relative to Hprt (hypoxanthine-guanine phosphoribosyltransferase). Data are presented as mean ± SEM, n = 10 mice per group (bleomycin treated) or 4 mice per group (saline control). Black bars: littermate control mice, open bars: myeloid specific Dnmt3b deficient mice.
